# Influence of Bulk-Fill Composites, Polimerization Modes, and Remaining Dentin Thickness on Intrapulpal Temperature Rise

**DOI:** 10.1155/2019/4250284

**Published:** 2019-12-04

**Authors:** Serdar Akarsu, Sultan Aktuğ Karademir

**Affiliations:** Department of Restorative Dentistry, Faculty of Dentistry, Ordu University, Ordu, Turkey

## Abstract

**Objectives:**

The aim of this study was to compare the effects of different bulk-fill resin composites, polimerization modes, and the thickness of remaining dentin on the increase of intrapulpal temperature.

**Methods:**

Human-extracted upper premolar teeth (*n* = 10) were used to design a single-tooth model with remaining dentin thicknesses of 1 mm and 0.5 mm. Estelite Bulk-fill Flow (Tokuyama, Japan), Surefil SDR™ Flow (Dentsply Caulk, Brazil), Filtek Bulk-Fill Posterior (3M, USA), and SonicFill™ 2 Bulk-fill (Kerr, USA) composites were applied according to the manufacturer's instructions. The standard and high modes of a light emitted diode (LED) light curing unit (LCU) (VALO™ Utradent, USA), were used for polymerization. In order to mimic the *in vivo* conditions of pulpal circulation, digital flowmetry (SK-600II, SK Medical, China) was used. Intrapulpal temperature rise was measured using K type thermocoupling (CEM DT 610B, Robosem Engineering, China). Data were analyzed using three-way variance analysis (ANOVA) and the independent *t*-test.

**Results:**

No significant statistical differences in intrapulpal temperature rise between low viscosity bulk-fill composites (SDR and Estelite) were found. The lowest intrapulpal temperature rise was found in groups which used the Filtek Bulk-fill composite. Decreases in the remaining dentin thickness increased the intrapulpal temperature rise.

**Significance:**

This study demonstrated that remaining dentin thickness, filler ratio of bulk-fill composites, and power and application time of the LED-LCU may affect intrapulpal temperature rise.

## 1. Introduction

Resin composites are widely used materials for various restorative processes. These materials, which were used in dentistry for esthetic restoration of anterior teeth in the early days, are now also widely used in posterior dental restorations due to the high demand for these procedures [1]. However, due to limited light penetration, there are disadvantages, such as the difficulty in polymerization of deep cavities [2]. The optimum method to overcome this problem is the application of an incremental technique in which polymerization is optimized for the resin composite [3]. The incremental technique, which has been widely used in the restoration of posterior teeth for some time, has some drawbacks such as, time requirements, the lack of space between the layers of the teeth, and the risk of contamination [4, 5].

In addition to optimizing the material used in the development of composite resins, it is particularly important to simplify the application. Recent improvements have led to the development of bulk-fill composites to reduce the disadvantages of the incremental technique [6]. Bulk-fill composites speed up restorative procedures because they can be applied in layers up to 4 mm, and in some circumstances, thicker in a single application [7, 8]. Some bulk-fill composites are produced as posterior restorative material, while others are produced only for use as base material that must be covered with a conventional resin composite [9]. Manufacturers claim that these materials show low polymerization shrinkage and an increased depth of curing [10, 11]. It was also reported that gap formation between composite layers and contamination are prevented [9, 11].

Today, due to their advantages such as long life, practicality, and short application time for the polymerization of resin composites, light-emitting diode (LED) light curing units (LCUs) with different light intensities are often preferred [12]. These light sources have different application modes developed to meet specific needs. LEDs may offer a variety of polymerization modes, including “soft start curing,” which may provide the resin composite with better viscoelastic flow during polymerization, thereby reducing shrinkage stress and microleakage [13]. Although it is thought that decreasing the light intensity decreases polymerization stress, it is also thought that the composite resin in the substrates cannot be polymerized sufficiently, affecting physical and mechanical properties negatively. LED-LCUs offer a narrow emission spectrum (a bandwidth of approximately 20 nm with a peak at 470 nm), and the spectrum falls closely within the absorption range of camphorquinone (CQ), the most frequently used photoinitiator in resin composites. In general, LED-LCUs have the following advantages: an extended lifetime of more than 10,000 hours, little light output degradation over time, and resistance to shock and vibration [14]. It is claimed that high-intensity LED-LCUs are able to reach irradiances of 2.000–3.200 mW/cm^2^ depending on the selected mode of use. New LED-LCUs emit lights with two or more different wavelength ranges (polywave). They produce both a shorter violet wavelength and a longer blue wavelength. Violet light is used to activate photoinitiators that are sensitive to light waves within the range of 350–420 nm in length. However, blue light activates photoinitiators, with maximum light absorbance close to 468 nm. Therefore, these polywave LED-LCUs are used to activate a wider range of photoinitiators. However, the different positions of light emitters along the same LCU tip could affect the homogeneity of the light output through the light guide tip [15]. Shorter irradiation periods are reportedly adequate for most of the recently introduced high-intensity LED-LCUs [14, 15].

Heat generation is the most severe form of stress that can occur to the pulp, and is caused by various operative procedures [16]. The pulp is a highly vascularized dental tissue and contains the main regulatory system for heat distribution in teeth, capable of dissipating the heat transferred by external thermal stimuli to the dentin-pulp complex. Conversely, it consists of a relatively large amount of tissue encased in dentin walls with a terminal circulation and no collateral blood supply. For this reason, pulp is susceptible to a rise in temperature when exposed to a thermal stimulus [17]. Although there is a lack of information regarding changes in pulp tissue under different curing protocols and applications, thermal reactions at the periphery of the pulp can cause injuries to the odontoblastic layer leading to degeneration, protoplasm coagulation, and expansion of liquid in the dentinal tubules [18]. Intrapulpal temperature rise during polymerization of resin composites is one of the most important conditions that may damage the pulp. Although the heat released during the exothermic reaction of composite resins may contribute to intrapulpal temperature rise, curing lights remain the most responsible heat source for this rise. Therefore, the curing light type, radiant exitance, radiant exposure values, and light beam profile play important roles in pulp temperature rise. The power and intensity of the light source, the remaining dentin thickness, the content of the resin composite, the placement technique of the resin composite (bulk-fill or incremental), and the distance between the light source and the resin composite are also factors that affect temperature rise [18].

Thermal trauma may occur due to cavity preparation and exothermic polymerization of resin-based restorative materials [19]. Various light sources are used for the polymerization of restorative materials [20], and if not controlled, may eventually damage the pulp tissue irreversibly [21].

The aim of this study was to compare the effects of different bulk-fill resin composites, polymerization modes, and thickness of remaining dentin upon intrapulpal temperature rise. The null hypotheses were as follows: (1) there are no differences in intrapulpal temperature rise during the light curing process among bulk-fill composites, and (2) the polymerization modes of VALO LED and the remaining dentin thickness do not produce significant differences in intrapulpal temperature rise.

## 2. Materials and Methods

This study was approved by the Ethics Committee of Ordu University, Turkey (2018-99) and supported by the Scientific Research Projects Coordination Unit (BAP) (A-1816). To determine the number of samples needed for the study, the G *∗* Power Software version 3.1.9.2 (Universität Düsseldorf, Germany) was used. During the polymerization of resin composites with similar properties, the sample size of a previous study evaluating intrapulpal temperature changes was used as a reference. A total of 144 samples were required in 16 groups, with a 95% confidence interval and 0.05 significance level. It was decided in the present study that 10 teeth would be used across 16 groups and therefore, the sample size was 160. Since a single-tooth model was used, all the materials were applied sequentially to each tooth.

**Figure 1:**
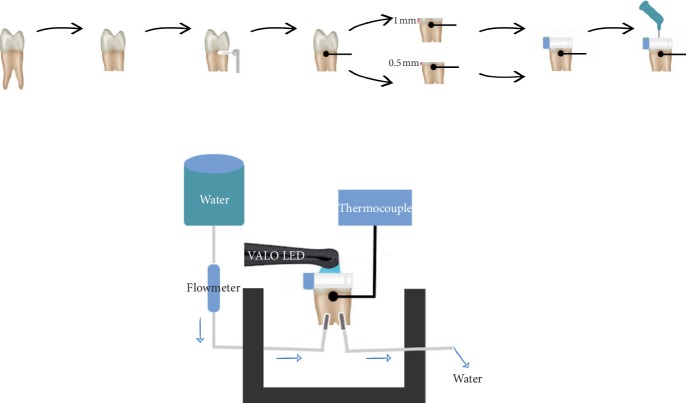
Schematic representation of intrapulpal temperature measurement.

In this *in vitro* study, 10 upper premolar teeth without caries and/or cracks, which were newly extracted for orthodontic reasons, were included. Remnants of periodontal tissues and debris on the teeth were removed with a pumice and brush. The teeth were stored in a distilled water solution at room temperature until further use. Roots were removed 5 mm below the cementoenamel junction using a water-cooled, low-speed diamond disk. The cementoenamel junction was prepared using a diamond fissure bur for placing a thermocouple. The K type thermocouple (CEM DT 610B, Robosem Engineering, China) could then make contact with the dentin wall on the roof of the pulp. Organic residues in the pulp chamber were removed using 5.25% sodium hypochlorite. The occlusal enamel and dentin were removed using a water-cooled diamond disk, 3 mm from the occlusal of the cementoenamel junction. Abrading was performed with 600-grit silicon carbide abrasive paper under running water to achieve a dentin thickness of 1 mm between the roof of the pulp and the occlusal dentin surface. The remaining dentin thickness was controlled by a precision caliper. In order to mimic the *in vivo* conditions of pulpal circulation, a fixed mechanism was developed to allow the physiological saline solution, at 36°C, to enter the pulp chamber at a flow rate of 0.026 mL/min from one side and exit again after circulation. The fluid pressure of the mechanism was adjusted to 20 cm H_2_O. A digital flow meter was used to control the flow rate (SK-600II infusion pump, SK Medical, Shenzhen, China). To measure the temperature increase in the pulp chamber, a K type thermocouple was used. The thermocouple tip was in contact with the dentin surface adjacent to the roof of the pulp. To ensure stability, the cavity at the cementoenamel junction was restored with Equia Forte (GC Corporation, Tokyo, Japan). Bulk-fill composite materials were placed on the occlusal surface of the tooth with a thickness of 4 mm using an Automatrix Band (Adapt SuperCap Matrix Kerr, Australia). The polymerization process was completed using the standard (1,000 mW/cm^2^, 20 sec) and high mode (1,440 mW/cm^2^, 12 sec) of the LED-LCU (VALO, Ultradent Inc, South Jordan, Utah, USA) device. For easy removal of bulk-fill composites after polymerization, no bonding agent was applied. The intrapulpal temperature was recorded before and after polymerization. Abrading was performed on the occlusal dentin surface with 600-grit silicon carbide abrasive paper under running water to achieve a dentin thickness of 0.5 mm. Using the 4 bulk-fill composites and VALO LED (standard and high mode), the increase in intrapulpal temperature was recalculated. The system of intrapulpal temperature measurement is schematically represented in Figure 1. The bulk-fill composite and product details are summarized in Table 1.

### 2.1. Statistical Evaluation

Statistical analysis was performed using the NCSS (Number Cruncher Statistical System) 2007 Statistical Software (Utah, USA) package. Descriptive statistics including means and standard deviations were calculated for each group. The Kolmogorov–Smirnov test and Levene's test were used to analyze whether the variables were evenly distributed. Analysis of intrapulpal temperature changes between the groups were calculated using ANOVA and independent *t*-tests.

## 3. Results

According to three-Way ANOVA, the differences between intrapulpal temperature increases were significant (Table 2).  Table 3 shows the mean and standard deviation values of the intrapulpal temperature rises.

In teeth where the thickness of the dentin was 1 mm, the highest increase in intrapulpal temperature was observed in the 1st group (5.76 ± 0.85), in which SDR bulk-fill composite was polymerized using the standard mode of VALO LED. The lowest temperature increase was observed in the 6th group (2.75 ± 0.69) where the Filtek bulk-fill composite was polymerized using the high mode of VALO LED. The intrapulpal temperature increase values (4.61–5.76) in teeth polymerized using the standard mode of VALO LED were higher than the intrapulpal temperature increase values (2.75–4.61) in teeth polymerized using the high mode.

In teeth where the thickness of the dentin was 0.5 mm, the highest intrapulpal temperature increase was observed in the 9th group (7.22 ± 1.17), in which the SDR bulk-fill composite was polymerized using the standard mode of VALO LED. The lowest intrapulpal temperature increase was observed in the 14th group (4.28 ± 0.74) where the Filtek bulk-fill composite was polymerized using the high mode of VALO LED. The intrapulpal temperature increase values (5.56–7.22) in teeth polymerized using the standard mode of VALO LED were higher than the intrapulpal temperature increase values (4.28–5.55) in teeth polymerized using the high mode.

In groups where both standard and high mode-polymerized composites were used, the decrease in dentin thickness was observed to further increase the intrapulpal temperature. In teeth with different dentin thicknesses, there were no significant differences in intrapulpal temperature increase (*P*=0.051) between the groups in which the SDR composite was polymerized in high mode. However, there was a significant difference between the other groups in terms of intrapulpal temperature increase (*P* < 0.05).

There was no significant statistical difference between low viscosity bulk-fill (SDR and Estelite) composites in terms of intrapulpal temperature increase.

## 4. Discussion

Polymerization of light-activated dental materials results in an increase in the heat released during the exothermic process, as well as an increase in the heat generated by the light source [22, 23]. The heat applied to tooth structures can cause different degrees of pulpal damage. Pohto and Scheinin reported that the critical temperature for the reversible damage of dental pulp was between 42°C and 42.5°C [24]. When the intrapulpal temperature rises to 5.5°C, the pulp may be irreversibly damaged [21]. Many studies have been conducted on intrapulpal temperature increase, and variable results have been reported. Factors that cause intrapulpal temperature to rise can be divided into three categories: factors related to the tooth (remaining dentin thickness, shade of dentin, and pulp microcirculation), factors related to the light source (wavelength, light intensity, and exposure time), and factors related to the properties of resin material (color and translucency/opacity) [25].

The remaining dentin thickness and thermal conductivity of the dentin are critical factors in the heat transfer to the pulp [26]. The present study is consistent with previous studies that show there is a relationship between the thickness of the remaining dentin and intrapulpal temperature increase [20, 27–30]. Previous studies on intrapulpal temperature change focused on the same group of teeth, while in others only one tooth was used [20, 31]. In the present study, 10 premolar teeth were used. Since a single tooth model was used, all materials and light device modes on each tooth were applied sequentially. When the dentin thickness decreased from 1 mm to 0.5 mm in all samples, intrapulpal temperature increased. Pulpal blood microcirculation is the main regulatory system for heat dissipation in teeth [32]. However, there is no consensus on pulpal blood flow. Baik et al. calculated the rate of pulpal blood flow [33] as 0.0026 ml/min. Therefore, in our study, water circulation was performed at a rate of 0.0026 ml/min to simulate *in vivo* conditions.

The light intensity of devices plays an important role in the intrapulpal temperature increase that occurs during polymerization of dental materials. High light intensity and duration of application increase intrapulpal temperature [11, 20, 34–36]. However, Ramoglu et al. reported that a certain period of time is necessary for the transmission of heat energy to the pulp, through enamel and dentin tissue. The short-time application of VALO LED, at high intensity could result in a lower intrapulpal temperature increase [37]. In agreement with the findings of Ramoğlu et al., our study showed that the intrapulpal temperature increase in teeth polymerized using the high mode of VALO LED was lower in both the samples with a 1 mm dentin thickness (2.75–4.61) and a 0.5 mm dentin thickness (4.28–5.55) than the intrapulpal temperature increase (1 mm dentin thickness: 4.61–5.76; 0.5 mm dentin thickness: 5.56–7.22) in teeth polymerized using the standard mode. This can be explained by the decrease in the application time of VALO LED (standard mode: 20 sec; high mode: 12 sec).

Composite resins generally contain a resin matrix, inorganic fillers, and a binding agent. Methacrylates, such as Bisphenol A-Glycidyl Methacrylate (Bis-GMA) and Urethane Dimethacrylate (UDMA), are used most commonly in composite materials. Triethylene glycol dimethacrylate (TEGDMA) is added to composite resins as a solvent [38]. Exothermic differences between different resin materials may be due to the type and amount of monomers present in the material, as well as the degree of conversion that occurs during the polymerization reaction [39]. The high conversion rate is characterized by increased heat [40]. Altintas et al. reported that methacrylate-based materials caused a statistically higher temperature increase, and that they would constitute a significant risk in cavities with dentin thickness of less than 1 mm [41]. When the chemical structures of the bulk-fill composites used in this study were examined, similar methacrylate monomer structures were observed, containing the following monomers: SDR (UDMA, ethoxylated bisphenol A dimethacrylate (EBPADMA), TEGDMA), Estelite bulk-fill (Bis-GMA, Bis-MPEPP, TEGDMA), Filtek bulk-fill, (Aromatic Urethane Dimethacrylate (AUDMA), UDMA, and 1, 12-dodecane-DMA monomers), and SonicFill (Bis-GMA, TEGDMA, EBPDMA).

It has been reported that when the filler content of resin composites is increased, the temperature rise decreases because the fillers are chemically inert and do not affect the reaction temperature [42]. However, fillers are capable of absorbing external energy. This energy can cause a temperature rise in the composite resin matrix. For this reason, the filler structure of the resin composite may play an indirect role in the temperature rise [43]. In the present study, the lowest intrapulpal temperature increase was observed for the Filtek bulk-fill composite, in both the different dentin thicknesses and polymerization modes (1 mm dentin thickness: 2.75–4.61; 0.5 mm dentin thickness: 4.28–5.56). The reason why Filtek bulk-fill (58.4%) and SonicFill composites produce a lower intrapulpal temperature increase may be due to the fact that they have a higher filler content than SDR (44%) and Estelite bulk-fill (56%) composites. Although the SonicFill has a higher filler ratio than the Filtek bulk-fill, lower intrapulpal temperature rise was not observed for Sonic fill compared with the Filtek bulk-fill composite. Due to the unique rheology of the Sonic fill composite, the application of sonic energy during restoration may facilitate the formation of a denser polymer network, which reduces the number of unreacted components. Exothermic heat, which occurs due to a more intense polymer network, can increase intrapulpal temperature.

Al Qudoah et al. reported that the temperature increases in the polymerization of light-colored composites begins after 5 sec, while the temperature increase in the polymerization of dark colored composites begins after 10 sec. The penetration of light reaches deeper in light-colored composites, whereas in dark-colored composites light penetration is more difficult [39]. Therefore, the same color (A2) was used in all materials used in this study.

Although the high mode of VALO LED caused lower intrapulpal temperature rise in this study, previous studies reported that the short-time application of high-intensity light curing has some disadvantages such as greater shrinkage stresses, a poorer interface, and more cytotoxicity [44].

This study has some important limitations such as the removal of the enamel and dentin walls, not applying an adhesive layer, applying resin composites at a 4 mm thickness, and measuring the intrapulpal temperature from a single point. In the presence of enamel and dentin walls, the increase in intrapulpal temperature may decrease due to heat dissipation. In addition, the adhesive layer may serve as a barrier to heat transfer under clinical conditions. In our study, 10 premolar teeth were used. Intrapulpal temperature increase values were measured using different composite and polymerization modes on the same teeth. Enamel and dentin walls were removed, and an adhesive layer was not applied in order to easily remove the composites during repeated measurements. In addition, composite thickness was determined as 4 mm according to the manufacturer's recommendations for obtaining standard intrapulpal measurements.

The null hypotheses in this study were rejected. There were significant differences in intrapulpal temperature rise during the light curing process between bulk-fill composites. The lowest intrapulpal temperature rises were observed in groups where the Filtek bulk-fill composite was polymerized using VALO LED. The polymerization modes of VALO LED and the remaining dentin thickness produced significant differences in intrapulpal temperature rise. The decrease in remaining dentin thickness was observed to further increase the intrapulpal temperature. The intrapulpal temperature rise in teeth polymerized using the high mode of VALO LED was lower than the intrapulpal temperature increase in teeth polymerized using the standard mode.

Although factors such as different types of bulk-fill composites, the remaining dentin thickness, and the polymerization modes of VALO LED were found to affect intrapulpal temperature rise, in order to evaluate clinical efficacy, our results should be evaluated in combination with those of other studies regarding the degree of conversion and microleakage and hardness, as well as intrapulpal temperature rise.

## 5. Conclusion

Within the limitations of this study, the remaining dentin thickness is an important factor in reducing intrapulpal temperature increase. Conservation of the affected dentin, as much as possible, during cavity preparation may result in a lower intrapulpal temperature increase. Using bulk-fill composites with a high filler ratio may reduce the intrapulpal temperature increase. The high mode of VALO LED may cause lower intrapulpal temperature rise than the standard mode.

## Figures and Tables

**Table 1:** Bulk-fill composites and the manufacturer's information.

Material/manufacturer	Monomers	Filler, particles sizes, percentage	Photoinitiators	Thickness/curing time and light intensity
SureFil® SDR + flow™ (DENTSPLY petropolis, Brazil)	Modified UDMA EBPADMA, TEGDMA	Barium and strontium fluoroalumino silicate glasses (4.2 *μ*m) 68 wt%, 44 vol%	Camphoroquinone, BHT, UV stabilizer, titanium dioxide, iron oxide pigments, fluorescing agent	4 mm/20 s 500–1,000 mW/cm^2^ (LED and halogen) or 10 s (high-power lights).
Estelite bulk-fill flow (Tokuyama, Japan)	Bis-GMA, Bis-MPEPP, TEGDMA	Supranano spherical filler (spherical SiO_2_-ZrO_2_) (200 nm) 70 wt%, 56 vol%	RAP technology™	4 mm/20 s 500–1,000 mW/cm^2^ (LED and halogen) or 10 s (high-power lights).
Filtek bulk™-fill posterior (3M, st. Paul, MN, USA)	Bis-GMA, AUDMA UDMA, DDDMA	Silica (20 nm), zirconia (4–11 nm), ytterbium trifluoride (100 nm), zirconia/silica–76.5 wt%, 58.4 vol%		4 mm/20 s ≥ 1,000 mW/cm^2^ (LED) or 40 s 550–1000 mW/cm^2^ (halogen light).
SonicFill™ (Kerr, CA, USA)	Bis-GMA, TEGDMA, EBPDMA	Nanoscale zirconium oxide, silica oxide particles (10–30 nm) 83.5 wt%, 69 vol%		5 mm/20 s 500–1,000 mW/cm^2^ (LED and halogen)

**Table 2:** Results of three-way analysis of variance with dependent variable (*C*).

Source of variation	Type III sum of squares	d*f*	Mean square	*F*	*P*
Polymerization	75,350	1	75,350	114,021	000
Bulk-fill composite	51,586	3	17,195	26,020	000
Dentin thickness	62,750	1	62,750	94,954	000
Polymerization *∗* bulk-fill composite	974	3	325	491	689
Polymerization *∗* dentin thickness	210	1	210	318	574
Bulk-fill composite *∗* dentin thickness	280	3	093	141	935
Polymerization *∗* bulk-fill composite *∗* dentin thickness	2,094	3	698	1,056	370
Error	95,162	144	661		
Total	4629,380	160			

**Table 3:** Mean and standard deviation values of the intrapulpal temperature rises.

Composite	VALO LED	Remaining dentin thickness of 1 mm		Remaining dentin thickness of 0.5 mm		*P* ^b^
		Mean	SD	Mean	SD	
SDR	Standard	5,76^C^	0,85	7,22^C^	1,17	0.005
	High	4,61^BC^	0,92	5,44^AB^	,84	0.051
ESTELITE	Standard	5,46^C^	0,95	7,05^C^	,90	0.001
	High	4,38^BC^	0,86	5,55^AB^	,93	<0.0001
FILTEK	Standard	4,61^BC^	0,88	5,56^AB^	,64	0.014
	High	2,75^A^	0,69	4,28^A^	,74	<0.0001
SONICFILL	Standard	5,10^BC^	0,59	6,40^BC^	,62	<0.0001
	High	3,99^AB^	0,52	5,18^AB^	,50	<0.0001
*P* ^a^		0,000	0,000			

^a^Results of one-way analysis of variance; ^b^results of independent *t*-test; SD indicates standard deviation; same superscript letters indicate not significantly different according to the Tukey test, *P* < 0.05. Different superscript letters indicate significantly different according to the Tukey test, *P* < 0.05.

## Data Availability

The data used to support the findings of this study are available from the corresponding author upon request.
